# Local and systemic safety of deproteinized calf blood extract injection: hypersensitivity, hemolysis, local tolerance, and acute intravenous toxicity in rodents and rabbits

**DOI:** 10.3389/fphar.2025.1709084

**Published:** 2026-01-05

**Authors:** Guodong Qin, Pengfei Zhao

**Affiliations:** 1 Department of Security, Guangdong Justice Police Vocational College, Guangzhou, China; 2 Department of Pharmacology, School of Pharmacy, China Medical University, Shenyang, China

**Keywords:** deproteinized calf blood extract, hemolysis, hypersensitivity, local tolerance, acute toxicity

## Abstract

**Introduction:**

Deproteinized calf blood extract injection (DCBEI) is widely applied in neurology and wound care, but systematic preclinical safety data are scarce. Because it is administered parenterally, assessment of immunogenicity, hemocompatibility, local tolerance, and acute toxicity is essential to meet regulatory standards.

**Methods:**

A focused safety package was developed, comprising: (i) active systemic anaphylaxis (ASA) in Hartley guinea pigs (n = 24, 4 groups); (ii) *in-vitro* hemolysis in rabbit erythrocytes, spectrophotometrically measured at 545 nm over 0.25–3 h; (iii) local tolerance in New Zealand White rabbits (n = 4 per regimen) after single or 6-day repeated intravenous (ear-vein) or intramuscular (quadriceps) dosing, assessed macroscopically and by histopathology; and (iv) acute intravenous toxicity in Kunming mice (n = 50, 5 groups, 3.075–6.150 g kg^-1^ total solids), with logistic regression used to estimate LD_50_ and 95% CI.

**Results:**

No DCBEI-treated animals developed ASA reactions ≥ grade 2 (0/6), while all ovalbumin controls showed severe grade 4 responses. Hemolysis remained ≤4.88% across all concentrations and timepoints, consistent with saline controls and below the 5% non-hemolytic threshold. Local tolerance was favorable, with macroscopic scores of 0 and no histopathological abnormalities after single or repeated administration. Acute dosing produced a dose-dependent mortality curve, yielding an LD_50_ of 4.35 g kg^-1^ (95% CI 4.03–4.69).

**Conclusion:**

This route-relevant dataset establishes a compact preclinical safety foundation for DCBEI, supporting its continued clinical use and guiding future investigations into long-term toxicology and translational risk management.

## 1 Introduction

Deproteinized calf blood extract (DCBE) is a biologically active product derived from the blood of calves aged 2–6 months, in which proteins are removed to yield a mixture of low–molecular-weight constituents, including peptides (<5 kDa), amino acids, nucleotides, oligosaccharides, lipids, and inorganic ions such as Na^+^, K^+^, Ca^2+^, and Mg^2+^ (Li et al., 2016; Qu et al., 2021; Jędrejko et al., 2023). This complex molecular composition supports pleiotropic biological functions: DCBE enhances mitochondrial uptake of oxygen and glucose, improves cellular metabolism under hypoxic conditions, stimulates ATP synthesis, and thereby promotes cell survival, proliferation, and tissue repair (Firan et al., 2020). These pleiotropic metabolic and neuroprotective effects have been consistently described in both preclinical and clinical contexts (Machicao et al., 2012).

Clinically, DCBE has been widely used in neurology and regenerative medicine. Reported benefits include symptomatic improvement in Alzheimer’s disease (Selezneva et al., 2023), cerebrovascular disorders (la Fleur et al., 2020), and neurological sequelae following stroke or traumatic brain injury (Firan et al., 2020). Preclinical findings further corroborate these effects: in a rat model of cerebral ischaemia, Actovegin treatment improved spatial learning and memory (Meilin et al., 2014), while the ARTEMIDA randomized controlled trial demonstrated cognitive benefit in post-stroke patients (Guekht et al., 2017). Beyond neurology, DCBE facilitates wound healing in chronic ulcers, traumatic lesions, and burns (Jędrejko et al., 2023; Li et al., 2020; Chuntrasakul, 1978; Chuntrasakul, 1984), and ophthalmic formulations have shown efficacy in ocular surface disease (Wang et al., 2022; Wu et al., 2021). Early administration has also been suggested to improve outcomes in neonatal hypoxic–ischemic encephalopathy (Li et al., 2021), and more recently, Actovegin has been evaluated in athletes for performance-related outcomes (Milovanović et al., 2024). Collectively, these studies highlight the broad therapeutic spectrum of DCBE. However, despite extensive clinical adoption, the parenteral safety profile of DCBE injection (DCBEI)—including immunogenicity, hemocompatibility, local tolerance, and acute systemic toxicity—remains insufficiently characterized.

Despite its wide spectrum of clinical applications, the safety profile of DCBE remains a matter of scientific and regulatory concern. Pharmacological studies have reported anti-inflammatory, antioxidant, and immunomodulatory effects (Qu et al., 2021; Firan et al., 2020), suggesting that DCBE can mitigate oxidative stress, attenuate inflammatory cascades, and modulate immune responses. While these properties extend its therapeutic promise, they do not substitute for systematic toxicological evaluation. Preclinical safety studies specifically addressing parenteral exposure—the predominant clinical route—are still scarce and fragmented. The novelty of our work lies in its holistic approach, as we integrate multiple safety endpoints into a single, unified evaluation, which is not only a novel method for DCBEI but also critical for advancing its clinical use and ensuring its safety in broader therapeutic applications.

Given the importance of safety in parenteral therapies, injectable biologics must undergo rigorous safety assessments, including hypersensitivity, hemocompatibility, local tissue tolerance, and acute systemic toxicity. These parameters are essential because infusion-related reactions, hemolysis, or injection-site irritation may compromise clinical utility and patient safety. International guidelines, such as ISO 10993 (e.g., ISO 10993–4: Interactions with Blood) and ICH M3, explicitly emphasize the need for hemocompatibility assays, acute systemic toxicity testing, and local tolerance evaluation in establishing a comprehensive safety profile for parenteral products (Committee for Medicinal Products for Human Use, 2009; Stang et al., 2014; CCS, 2017). For deproteinized calf blood extract injection (DCBEI), however, available data are piecemeal and lack an integrated, route-relevant preclinical safety assessment.

In this context, the present study was designed to provide a concise yet comprehensive preclinical safety evaluation of DCBEI with a focus on parenteral use. Four key endpoints were investigated: (i) active systemic anaphylaxis (ASA) in guinea pigs to assess acute hypersensitivity potential; (ii) *in-vitro* hemolysis using rabbit erythrocytes to evaluate blood compatibility; (iii) local tolerance in rabbits following intravenous and intramuscular dosing to detect macroscopic and histological signs of irritation; and (iv) acute intravenous toxicity in mice to establish an LD_50_ and define systemic safety margins. Together, these tests address the most critical aspects of injection-related safety and align with current regulatory expectations for biologics.

**FIGURE 1:**
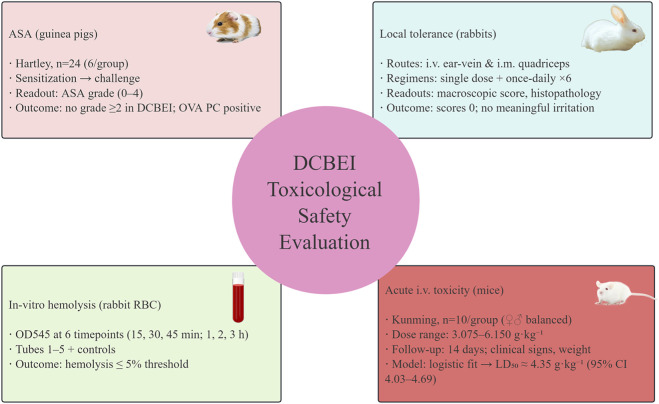
Study design. Schematic of four endpoints: ASA (guinea pigs), *in-vitro* hemolysis, local tolerance (single and 6-day repeated i. v./i.m. in rabbits), and acute i. v. toxicity in mice; timelines and sampling.

By integrating results across these endpoints, our work defines a foundational safety package for DCBEI. This integrated approach is the first of its kind for DCBEI injection, providing a holistic safety assessment in line with current regulatory expectations. It fills a critical gap in the literature, offering the first systematic evaluation of DCBEI’s safety profile for parenteral use. These findings not only support its continued clinical use but also provide guidance for optimizing administration practices, such as dilution and infusion rates, to mitigate potential risks. In doing so, this study contributes to the broader objective of ensuring that injectable biologics like DCBEI can be used safely and effectively in diverse therapeutic settings. The overall study design is illustrated in Figure 1.

## 2 Methods

### 2.1 Test item and controls

Deproteinized calf blood extract injection (DCBEI) was obtained from Shenyang Wanhe United Pharmaceutical Co., Ltd. (commercial formulation, 20 mL per vial, containing 800 mg total solids; batch no. 20250601). The labeled vehicle was water for injection. For the acute toxicity study, a 10-fold concentrate of DCBEI (total solids 410 mg mL^-1^, free amino acids 11 mg mL^-1^) was provided by the same manufacturer and diluted with 0.9% sodium chloride solution (25A19C13) to achieve the designated dose levels. Negative controls consisted of 0.9% sodium chloride injection (25A19C13, Parenteral tests) and isotonic saline (25A19C13, Hemolysis assays). Distilled water was used as the positive control for hemolysis (100% hemolysis reference). For the active systemic anaphylaxis (ASA) assay, 1% ovalbumin (Sigma, A5253) served as the positive control. The selection of test items and controls followed international standards for hemocompatibility and parenteral safety testing (International Organization for Standardization, 2017; NMPA, 2005).

### 2.2 Animals and housing

Hartley guinea pigs (male, 10 weeks old, 300–400 g; n = 24, 6 per group) were used for active systemic anaphylaxis (ASA) testing. New Zealand White rabbits (3 months old, 2–3 kg, sex balanced) were employed for local tolerance evaluation. Eight rabbits received a single-dose administration (intravenous ear vein or intramuscular quadriceps, n = 4 each), and an additional eight rabbits received repeated administration once daily for six consecutive days (intravenous or intramuscular, n = 4 each). Kunming mice (18–22 g; n = 50, 10 per group, sex balanced) were used for acute intravenous toxicity testing.

All animals were housed under specific pathogen–free (SPF) conditions with controlled temperature (20 °C–25 °C), relative humidity (30%–70%), and a 12 h light/dark cycle, with *ad libitum* access to standard chow and water. All procedures were reviewed and approved by the Institutional Animal Care and Use Committee of China Medical University and conducted in accordance with ARRIVE 2.0 guidelines.

### 2.3 Active systemic anaphylaxis (ASA)

Rationale. ASA testing in guinea pigs is a standard model to evaluate whether parenteral products can trigger IgE-mediated hypersensitivity reactions. This assay is recommended for biologics containing protein- or peptide-derived components that may provoke immune responses (Verdier et al., 1994; NMPA, 2005).

Procedure. Hartley guinea pigs (n = 24, 6 per group) were randomly allocated to four groups: saline (negative control), 1% ovalbumin (positive control), DCBEI low dose, and DCBEI high dose. Animals were sensitized by intraperitoneal injection on days 0, 2, and 4 with 1.85 mL kg^-1^ of saline, ovalbumin (1%), DCBEI 0.925 mL kg^-1^ (low dose), or DCBEI 1.85 mL kg^-1^ (high dose). On day 18, all groups were challenged intravenously (saphenous vein) with twice the sensitization dose (2× volume).

Clinical signs were observed for 3 h and graded on a 0–4 scale according to Supplementary Table S6; grade ≥2 was considered positive for hypersensitivity. The guinea pig ASA model has been validated against panels of reference compounds and is recognized by regulatory guidance (Verdier et al., 1994; NMPA, 2005).

### 2.4 *In vitro* hemolysis assay

Rationale. Hemolysis testing evaluates whether injectable formulations induce erythrocyte lysis and is required under ISO 10993–4 for hemocompatibility. A hemolysis rate >5% is considered unacceptable for parenteral products. The thresholds and procedures used here follow international standards and prior studies (Weber et al., 2018; Seyfert et al., 2002; Stang et al., 2014; Dobrovolskaia et al., 2008), as well as the Chinese NMPA technical guidelines (NMPA, 2005).

Preparation of erythrocyte suspension. 20 mL of rabbit blood was collected and placed into a conical flask containing glass beads. The blood was stirred with a glass rod for 5 min to remove fibrinogen, resulting in defibrinated blood. Five milliliters of the defibrinated blood was then mixed with 0.9% sodium chloride injection to a final volume of 10 mL and thoroughly mixed. The mixture was centrifuged in two tubes at 1,000 rpm for 15 min. The supernatant was discarded, and the pellet of erythrocytes was washed 2–3 times with 0.9% sodium chloride injection by repeating the centrifugation step, until the supernatant was clear. The final erythrocyte pellet was resuspended in 0.9% sodium chloride injection to prepare a 2% erythrocyte suspension, which was then used for the experiments.

Procedure. A 2% erythrocyte suspension was prepared from defibrinated rabbit blood by repeated washing in 0.9% sodium chloride until the supernatant was clear. For each timepoint, seven tubes were prepared: DCBEI serial dilutions (tubes 1–5), negative control (0.9% NaCl, tube 6), and positive control (distilled water, tube 7). Samples were incubated at 37 °C ± 0.5 °C and assessed at 15, 30, 45, 60, 120, and 180 min. After centrifugation, supernatants were analyzed spectrophotometrically at 545 nm. As detailed in Supplementary Table S7, the sample addition scheme for the *in vitro* hemolysis test of DCBEI is outlined.

Hemolysis (%) was calculated as: $\text{Hemolysis }\left(\%\right)=\frac{OD_{\text{test}}‐OD_{\text{nc}}‐OD_{\text{bg}}}{OD_{\text{pc}}‐OD_{\text{nc}}}\times 100\%$ , where OD_test_ = absorbance of test tube, OD_nc_ = negative control, OD_pc_ = positive control, and OD_bg_ = background absorbance of DCBEI at the same dilution. A threshold of >5% was considered hemolytic and non-acceptable.

### 2.5 Local tolerance in rabbits

Rationale. Local tolerance testing is a critical requirement for injectable biologics to identify potential vascular or muscular irritation at the injection site. Such evaluations are emphasized in both ICH guidance and EMA/Chinese pharmacopoeial guidelines (European Medicines Agency, 2015; NMPA, 2005).

Procedure. New Zealand White rabbits were allocated into single-dose (n = 8; i. v. ear vein or i. m. quadriceps, n = 4 each) and repeated-dose groups (n = 8; daily dosing ×6; i. v. or i. m., n = 4 each). For intravenous dosing, 0.5 mL of DCBEI was injected into the left ear vein, with 0.9% sodium chloride administered into the contralateral ear vein as a paired control. For intramuscular dosing, 0.5 mL of DCBEI was administered into the left quadriceps, with saline in the contralateral muscle.

Macroscopic evaluation. Injection sites were observed daily and scored for erythema, swelling, or necrosis using validated scales for skin/muscle irritation (Supplementary Tables S8–S10). An average score ≥0.5 (i.v.) or ≥2 (i.m.) was predefined as unacceptable.

Histopathology. At 96 h after the final administration, animals were euthanized and injection-site tissues were collected, fixed in 10% neutral-buffered formalin, and examined by H&E staining. Abnormal findings such as thrombus formation, endothelial disruption, edema, inflammatory infiltrates, necrosis, or fibroplasia were considered evidence of irritation. Remaining animals were observed up to 14 days to assess reversibility of any lesions.

This study design follows internationally recognized rabbit models of local tolerance for injectable products (Segal et al., 2015; European Medicines Agency, 2015).

### 2.6 Acute intravenous toxicity in mice

Rationale. Determining acute intravenous toxicity and establishing the LD_50_ is a fundamental component of preclinical safety evaluation. This provides an estimate of systemic tolerance and safety margin for parenteral products, in line with OECD and ICH recommendations (Bruce, 1985; Lipnick et al., 1995).

Procedure. Fifty Kunming mice (18–22 g; n = 10 per group, sex-balanced) were randomized into five dose groups: 3,075, 3,650, 4,346, 5,169, and 6,150 mg kg^-1^ (total solids, i. v. tail vein). The injection volume was 0.3 mL per 10 g body weight, administered at 1 mL min^-1^, consistent with clinical infusion parameters scaled to mice. After dosing, animals were observed for acute clinical signs, and mortality was recorded over 14 days. Dead animals were immediately necropsied for gross pathology, with histopathology performed when indicated.

Analysis. Dose–mortality data were modeled using binomial logistic regression with log_10_ (dose) as the predictor. The LD_50_ and 95% confidence interval were estimated from the fitted curve. This design, with five dose groups of 10 animals each, conforms to established acute toxicity testing standards. While alternative validated approaches (e.g., up-and-down procedures) can reduce animal use, prior studies confirm that comparable LD_50_ estimates are obtained (Bruce, 1985; Lipnick et al., 1995), supporting the adequacy of the chosen sample size.

### 2.7 Statistics

Continuous variables are expressed as mean ± standard deviation (SD) unless otherwise specified. The LD_50_ was estimated by binomial logistic regression of mortality versus log_10_ (dose) (five dose levels, n = 10 per group), providing both point estimate and 95% confidence interval. Group comparisons for categorical proportions were conducted using Fisher’s exact test. A two-sided significance level of α = 0.05 was applied, with multiplicity adjustment performed using the Holm-Šidák method where applicable. Statistical analyses were carried out with GraphPad Prism 10.2 (GraphPad Software, San Diego, CA) and R version 4.3.2 (R Foundation for Statistical Computing, Vienna, Austria).

## 3 Results

### 3.1 Active systemic anaphylaxis

At the challenge phase on day 14, all guinea pigs in the ovalbumin positive control group (6/6) developed severe grade 4 reactions, characterized by convulsions, respiratory failure, and shock, consistent with a strong IgE-mediated response. In contrast, no animal treated with DCBEI (low- or high-dose) or saline control exhibited systemic reactions of grade ≥2 (0/6 per group). Individual scoring outcomes are provided in Supplementary Table S1.

Based on the FDA’s weight-of-evidence framework for immunotoxicity testing (U.S. Food and Drug Administration, 2023), and the absence of both biological triggers and ASA positivity, additional follow-up studies [e.g., T-cell dependent antibody response or specialized immunotoxicology assays] were not warranted. These results support a lack of systemic hypersensitivity potential for DCBEI under the tested conditions.

### 3.2 *In vitro* hemolysis

Visual inspection. At all observation points (15–180 min), test tubes 1–6 (DCBEI and saline) showed complete sedimentation of erythrocytes with clear, colorless supernatants, without red or brown flocculent precipitates. In contrast, tube 7 (distilled water) presented bright red transparent supernatants without residual cells, consistent with complete hemolysis.

**FIGURE 2:**
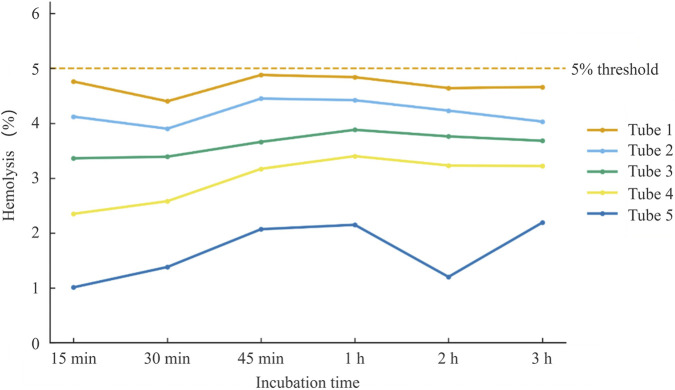
*In vitro* hemolysis of DCBEI in rabbit erythrocytes. Serial additions in tubes 1–5 were incubated at 37 °C and absorbance was measured at 545 nm after 15, 30, and 45 min, and 1, 2, and 3 h. Hemolysis remained ≤4.88% under all conditions, below the 5% non-hemolytic threshold (dashed line). Saline control showed ≈0% hemolysis, whereas distilled water reached 100% hemolysis (not plotted). Data are presented as connected lines; error bars not shown.

Spectrophotometric assay. OD_545_ absorbance measurements (Supplementary Table S2) confirmed the visual findings. Across all test concentrations and incubation intervals, DCBEI induced hemolysis rates ≤4.88%. Saline controls showed negligible hemolysis (∼0%), whereas distilled water yielded 100% hemolysis. These results are illustrated in Figure 2, which demonstrates that DCBEI consistently remained below the 5% non-hemolytic threshold, aligning with ISO 10993–4 criteria and previous ASTM-aligned studies (International Organization for Standardization, 2017; Dobrovolskaia et al., 2008).

### 3.3 Local tolerance

Intravenous administration. Macroscopic observations at 1, 24, 48, 72, and 96 h after single dosing, as well as before each dose and up to 96 h after the final injection in the 6-day repeated regimen, revealed no visible irritation at the injection sites. Histopathological evaluation confirmed intact vascular endothelium, with no evidence of thrombosis, edema, inflammatory infiltration, or necrosis.

Intramuscular administration. Similarly, no visible irritation was observed after single or repeated intramuscular dosing, as assessed at 1–96 h post-injection. Histopathological examination of quadriceps tissue at 96 h showed preserved muscle fibers without edema, necrosis, inflammatory infiltration, or fibroplasia.

**FIGURE 3:**
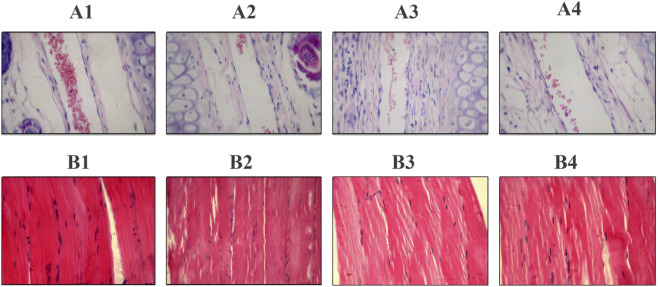
Local tolerance. Representative H&E-stained tissue sections from the injection sites (vascular or muscle tissues) at 96 h (400×). Intravenous (IV): Deproteinized Calf Blood Extract Injection (DCBEI), single dose (A1) and repeated doses ×6 (A2); 0.9% Sodium Chloride Injection, single dose (A3) and repeated doses ×6 (A4). Intramuscular (IM): DCBEI, single dose (B1) and repeated doses ×6 (B2); 0.9% Sodium Chloride Injection, single dose (B3) and repeated doses ×6 (B4).

Quantitative macroscopic and histopathological scores are provided in Supplementary Table S3 (i.v.) and Supplementary Table S4 (i.m.). Representative H&E sections are presented in Figure 3, confirming intact vascular and muscular structures. Local tolerance study design and interpretation adhered to the NMPA guideline on irritation, allergy, and hemolysis testing (NMPA, 2005) and the EMA Guideline on Non-clinical Local Tolerance Testing of Medicinal Products (European Medicines Agency, 2015).

### 3.4 Acute intravenous toxicity and LD_50_

**FIGURE 4:**
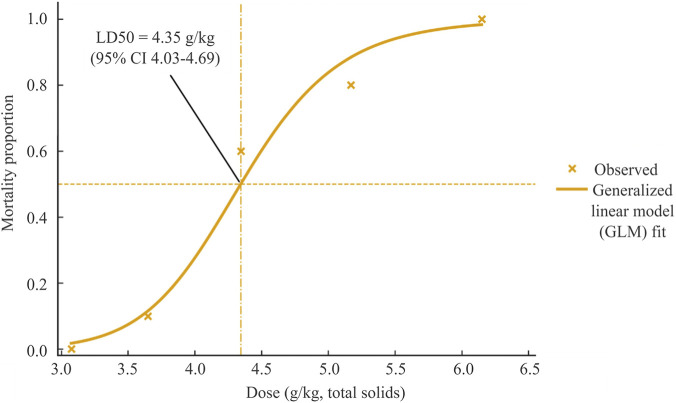
Acute intravenous toxicity of DCBEI in mice: dose–mortality curve and LD_50_ estimate. Kunming mice (n = 10 per group; sex-balanced) received tail-vein doses of 3.075–6.150 g kg^-1^ (total solids) and were monitored for 14 days. Dots represent observed mortality proportions, the solid curve depicts the binomial logistic regression with log_10_-dose as predictor, and the dashed vertical line indicates the LD_50_. The estimated LD_50_ was 4.35 g kg^-1^ (95% CI, 4.03–4.69 g kg^-1^).

Mortality in mice increased in a dose-dependent manner: 0/10 (3.075 g kg^-1^), 1/10 (3.650 g kg^-1^), 6/10 (4.346 g kg^-1^), 8/10 (5.169 g kg^-1^), and 10/10 (6.150 g kg^-1^). Detailed mortality counts are provided in Supplementary Table S5. Logistic regression on log_10_ (dose) yielded an estimated LD_50_ of 4.35 g kg^-1^ (95% CI: 4.03–4.69), as shown in Figure 4.

Surviving animals displayed transient signs (ataxia, shallow respiration, tachycardia) that resolved within 1–2 h, after which normal feeding, grooming, and activity resumed. Over the 14-day observation period, no delayed mortality or gross pathological abnormalities were observed. Body weight changes are shown in Supplementary Table S11 and Supplementary Figure S1. Necropsy revealed no gross lesions in major organs.

Human equivalent dose (HED). Based on FDA-recommended K_m_ conversion (mouse K_m_ = 3; human K_m_ = 37), the LD_50_ corresponds to an HED of ∼353 mg kg^-1^, equivalent to ∼21.2 g for a 60 kg adult. This contrasts with earlier estimates using a simplified factor of 9, which underestimated the HED.

Infusion-rate comparison. In this study, mice received tail-vein injections within 1 min. Scaling to humans, the HED equates to ∼21.2 g/min, whereas the maximum clinical infusion rate is ∼36.4 mg/min (400 mg over 11 min). Thus, the experimental injection rate was approximately 582-fold (≈10^3^) higher than clinical conditions, highlighting the conservative safety margin.

## 4 Discussion

This study provides one of the first route-aligned preclinical safety packages for deproteinized calf blood extract injection (DCBEI). The experimental design covered four complementary endpoints—active systemic anaphylaxis (ASA), hemolysis, local tolerance, and acute intravenous toxicity—thus enabling a compact yet comprehensive appraisal of acute and local risks associated with parenteral use. Collectively, our findings indicate that DCBEI exhibits a favorable acute safety profile within clinically relevant exposure ranges.

### 4.1 Interpretation of results in context

The absence of ASA responses in guinea pigs is notable, as biologics containing peptide or protein components often carry risks of IgE-mediated hypersensitivity. The ovalbumin control elicited severe reactions, validating the model, whereas DCBEI induced no grade ≥2 responses, consistent with clinical reports where infusion-related hypersensitivity is rarely observed (Qu et al., 2021). These findings align well with previous research on DCBEI’s clinical applications. For example, Qu et al. (2021) demonstrated that DCBEI mitigates neurodegeneration in Alzheimer’s disease, and Firan et al. (2020) have highlighted its regenerative properties in wound healing. By providing one of the first route-aligned safety packages, our study supports these findings and adds rigor by systematically evaluating critical safety parameters, such as hemolysis, local tolerance, and acute toxicity. This study is the first to provide a comprehensive preclinical safety evaluation of DCBEI injection, addressing all key aspects of parenteral safety in a single study. By combining hypersensitivity, hemolysis, local tolerance, and acute toxicity assessments, we provide a holistic view of DCBEI’s safety, a key advancement over fragmented prior reports. This further corroborates its potential for clinical use across various therapeutic settings, including neurology and regenerative medicine. Given the established clinical applications of DCBEI, including stroke rehabilitation and wound healing, these preclinical findings underscore the clinical potential of DCBEI in neurological rehabilitation, traumatic brain injury, and chronic wound care.

In the hemolysis assay, DCBEI produced ≤4.88% hemolysis at all concentrations and timepoints (15–180 min), remaining below the 5% non-hemolytic threshold defined by ISO 10993–4. These data confirm that DCBEI is compatible with erythrocytes, a critical prerequisite for intravenous products, in line with reports of other parenteral biologics (Weber et al., 2018).

Local tolerance studies in rabbits also supported compatibility. No irritation or histopathological injury was observed after single or repeated intravenous or intramuscular dosing. These data meet EMA guidance for local tolerance testing (European Medicines Agency, 2015) and corroborate prior reports that DCBEI promotes tissue repair without causing local adverse effects (Firan et al., 2020).

Acute intravenous toxicity studies revealed a clear dose–mortality relationship, with an LD50 of 4.35 g kg^-1^ (95% CI: 4.03–4.69 g kg^-1^). This tolerance margin is far above therapeutic exposure ranges, providing reassurance about systemic safety.

### 4.2 Mechanistic considerations

The clinical signs observed in non-surviving mice—including coma, respiratory depression, tachycardia, and urinary incontinence—were not consistent with DCBEI’s known adverse event profile. Instead, these manifestations align with acute ethanol intoxication, which is characterized by initial excitation followed by CNS depression, culminating in respiratory arrest at sufficiently high blood concentrations. Calculations from our dosing regimen suggested that blood ethanol concentrations reached ∼22,880–38,550 mg/L in higher dose groups, greatly exceeding the lethal threshold for rodents and humans (≈4,000 mg/L) (Kalant, 1996; Jones, 2019).

Further, confirmatory injections of saline solutions containing equivalent ethanol dilutions reproduced comparable neurotoxic signs, reinforcing the interpretation that vehicle excipients, rather than the active fraction, were the primary drivers of lethality. DCBEI formulations contain ∼40% ethanol and ∼40% propylene glycol; both solvents are widely used in parenteral preparations but are known to induce CNS depression at supratherapeutic levels (Arulanantham and Genel, 1978; Wilson et al., 2005). Ethanol is classified as a mild toxin with dose-dependent CNS effects, while propylene glycol is generally considered safe but can cause acidosis, seizures, and CNS depression at high exposure (Lim et al., 2014).

Taken together, the observed LD_50_ likely reflects excipient overload during rapid bolus administration rather than inherent toxicity of DCBEI’s bioactive small-molecule fraction. This underscores the importance of evaluating formulation excipients in preclinical studies and adjusting infusion parameters (e.g., dilution, infusion rate) to mitigate solvent-driven toxicity.

### 4.3 Clinical and translational relevance

For parenteral biologics, hemocompatibility and local tolerability are central determinants of infusion-related safety. Our results demonstrate that DCBEI can be administered intravenously or intramuscularly without acute concerns regarding red cell compatibility or local tissue injury. These findings provide preclinical reassurance that complements its established clinical applications in stroke rehabilitation, traumatic brain injury, wound healing, and regenerative settings (Firan et al., 2020; Chuntrasakul, 1978; La Fleur et al., 2022). The preclinical safety profile of DCBEI demonstrates its compatibility with intravenous and intramuscular administration, making it suitable for a wide array of clinical applications. Notably, its neuroprotective and wound-healing effects, as demonstrated in clinical studies (Firan et al., 2020; La Fleur et al., 2022), suggest its potential role in neurological rehabilitation, traumatic brain injury, and chronic wound care. Moving forward, more targeted clinical trials in these areas could further validate DCBEI’s therapeutic benefits, particularly in conditions requiring parenteral treatment. Given its favorable safety profile, DCBEI could be a novel therapeutic option for conditions such as Alzheimer’s disease and ischemic stroke, where few treatments are available.

Beyond confirming compatibility, the results also inform practical risk mitigation strategies. Although DCBEI itself showed negligible risk of hypersensitivity or hemolysis in standardized models, infusion-related events in clinical practice may still arise from vehicle excipients (e.g., ethanol, propylene glycol), infusion rates, or concomitant therapies. Accordingly, measures such as adequate dilution, controlled infusion speed, and close monitoring of patients with prior allergic histories remain advisable. These considerations mirror established recommendations for other parenteral biologics and are consistent with regulatory guidance that emphasizes infusion management as part of risk minimization (ISO 10993–4:2017; Committee for Medicinal Products for Human Use, 2009).

Collectively, the translational implication is that DCBEI possesses a favorable acute safety margin under controlled conditions, but responsible administration practices are critical to maintaining safety in real-world use. This underscores the importance of integrating preclinical findings with clinical vigilance to ensure optimal patient outcomes.

### 4.4 Strengths and limitations

A key strength of this work is its integrated, cross-endpoint design, which consolidated multiple acute safety parameters—ASA, hemolysis, local tolerance, and acute toxicity—into a single study framework. By applying standardized animal models and harmonized readouts, we generated a coherent and route-aligned safety profile of DCBEI, providing regulatory-relevant evidence without fragmenting assessments across separate studies. This compact design enhances interpretability and facilitates translational insights for clinical practice.

Several limitations warrant consideration. First, hemolysis testing was limited to short incubation windows (≤3 h), precluding detection of delayed red cell effects. Complement activation, platelet aggregation, and cytokine release—known contributors to infusion-related reactions—were not evaluated. Second, systemic toxicity assessment was confined to a single-dose acute model. Sub-acute and chronic repeat-dose studies are necessary to establish no-observed-adverse-effect levels (NOAELs) and to identify potential target organs. Third, we did not investigate reproductive or developmental toxicity, pharmacokinetics, or accumulation of residual proteins, which remain relevant for comprehensive risk assessment of biologics. Finally, the experimental design did not address inter-individual variability in immune reactivity or metabolism, which may influence clinical outcomes.

Together, these caveats emphasize that while the present findings support the acute parenteral safety of DCBEI, additional studies—particularly long-term, mechanistic, and translational evaluations—are essential to complete its preclinical safety dossier.

### 4.5 Future directions

Future research should broaden the safety evaluation of DCBEI beyond the acute setting. Hemocompatibility testing needs to be expanded to include full ISO 10993–4 endpoints such as complement activation, platelet aggregation, and coagulation parameters, which together capture a more comprehensive profile of blood compatibility. Sub-acute (14–28 days) and chronic (90 days) repeat-dose toxicology studies are required to define no-observed-adverse-effect levels (NOAELs), identify target organs, and characterize systemic tolerance under conditions that mimic sustained clinical exposure. In parallel, the integration of immunotoxicology endpoints—including cytokine release assays, T-cell activation, and assessment of delayed hypersensitivity—will further strengthen risk characterization. Studies in larger animal models using infusion regimens aligned with human clinical practice could enhance translational relevance by better replicating pharmacokinetics and exposure dynamics. Given the promising preclinical results, future studies should focus on long-term toxicological assessments, including sub-acute and chronic repeat-dose studies. It would be beneficial to expand testing using larger animal models, such as rats or non-human primates, to better mimic human responses and refine the human-equivalent dose (HED) calculations. Additionally, integrating immunotoxicology endpoints, such as T-cell activation and cytokine release assays, will help further define the immune response to DCBEI and refine safety protocols for human trials.

From a translational perspective, coupling preclinical toxicology with real-world pharmacovigilance is essential. Observational registries and prospective clinical safety monitoring can validate the low incidence of hypersensitivity and infusion-related reactions suggested by this preclinical package. Together, these approaches will refine evidence-based dosing guidelines and reinforce the regulatory foundation for DCBEI in neurological rehabilitation, wound repair, and other therapeutic contexts.

## 5 Conclusion

In conclusion, this preclinical study establishes a route-aligned safety profile for deproteinized calf blood extract injection (DCBEI). Across four complementary endpoints, DCBEI showed no evidence of acute immunogenicity in guinea pigs, favorable hemocompatibility *in vitro*, good local tolerance in rabbits, and a wide systemic safety margin in mice. Collectively, these findings provide a robust non-clinical foundation supporting its continued therapeutic use in neurological rehabilitation, wound repair, and related indications. Our study provides the first comprehensive preclinical safety profile for DCBEI injection, covering critical aspects of parenteral safety. Our findings support its continued clinical use and lay the groundwork for future regulatory submissions and clinical trials, particularly in neurology and regenerative medicine. At the same time, the results delineate clear priorities for further investigation—particularly extended repeat-dose toxicology, immunotoxicity profiling, and translational infusion-regimen studies—to ensure comprehensive risk characterization and regulatory alignment.

## Data Availability

The original contributions presented in the study are included in the article/Supplementary Material, further inquiries can be directed to the corresponding author.
