# Supplementary material for: Local and systemic safety of deproteinized calf blood extract injection: hypersensitivity, hemolysis, local tolerance, and acute intravenous toxicity in rodents and rabbits
Source: Front Pharmacol. 2026 Jan 5;16:1709084. doi: 10.3389/fphar.2025.1709084 (PMC12813156; doi:10.3389/fphar.2025.1709084)
Supplement: Supplementary file 1 [file Table1.docx]

**Supplementary Table S1. Active Sensitization Test Results of Deproteinized Calf Blood Extract Injection (expressed as reaction grades, n = 6).**

| Positive  Control Group | Negative  Control Group | Deproteinized Calf Blood Extract Injection (Low-Dose Group) | Deproteinized Calf Blood Extract Injection (High-Dose Group) |
| --- | --- | --- | --- |
| 4.000.00 | 0.000.00 | 0.000.00*^△^ | 0.000.00*^△^ |

*** P < 0.05 vs. Positive Control Group; △ P > 0.05 vs. Negative Control Group.**

**Supplementary Table S2. Spectrophotometric Assessment of In Vitro Hemolysis Induced by Deproteinized Calf Blood Extract Injection Injection.**

| **Observation Time OD Value and Hemolysis Rate** | | **Tube Number** | | | | | | |
| --- | --- | --- | --- | --- | --- | --- | --- | --- |
|  |  | **1** | **2** | **3** | **4** | **5** | **6** | **7** |
| 15min | OD | 0.100 | 0.090 | 0.078 | 0.062 | 0.041 | 0.025 | 1.602 |
|  | Hemolysis Rate % | 4.76 | 4.12. | 3.36 | 2.35 | 1.01 | 0 | — |
| 30min | OD | 0.101 | 0.093 | 0.085 | 0.072 | 0.053 | 0.031 | 1.622 |
|  | Hemolysis Rate % | 4.40 | 3.90 | 3.39 | 2.58 | 1.38 | 0 | — |
| 45min | OD | 0.112 | 0.105 | 0.092 | 0.084 | 0.066 | 0.032 | 1.673 |
|  | Hemolysis Rate % | 4.88 | 4.45 | 3.66 | 3.17 | 2.07 | 0 | — |
| 1h | OD | 0.115 | 0.108 | 0.099 | 0.091 | 0.070 | 0.034 | 1.709 |
|  | Hemolysis Rate % | 4.84 | 4.42 | 3.8806 | 3.403 | 2.1493 | 0 | — |
| 2h | OD | 0.119 | 0.112 | 0.104 | 0.095 | 0.074 | 0.040 | 1.741 |
|  | Hemolysis Rate % | 4.64 | 4.23 | 3.76 | 3.23 | 1.20 | 0 | — |
| 3h | OD | 0.129 | 0.118 | 0.112 | 0.104 | 0.086 | 0.048 | 1.786 |
|  | Hemolysis Rate % | 4.66 | 4.03 | 3.68 | 3.22 | 2.19 | 0 | — |

**Note:** Tube 6 served as the saline control, and Tube 7 served as the distilled water control.

**Supplementary Table S3. Macroscopic Irritation Reactions after Single and Repeated Intravenous Administration of Deproteinized Calf Blood Extract Injection (96 h, n = 4).**

| Group | 1 | |  | 2 | |  | 3 | |  | 4 | |
| --- | --- | --- | --- | --- | --- | --- | --- | --- | --- | --- | --- |
|  | Left Ear | Right Ear |  | Left Ear | Right Ear |  | Left Ear | Right Ear |  | Left Ear | Right Ear |
| Single Intravenous Injection | 0 | 0 |  | 0 | 0 |  | 0 | 0 |  | 0 | 0 |
| Repeated Intravenous Injection (×6) | 0 | 0 |  | 0 | 0 |  | 0 | 0 |  | 0 | 0 |

Note: The Left Ear served as the experimental site and received 0.5 mL of diluted Deproteinized Calf Blood Extract Injection, while the Right Ear served as the control site and received 0.5 mL of 0.9% Sodium Chloride Injection.

**Supplementary Table S4. Macroscopic Irritation Reactions after Single and Repeated Intramuscular Administration of Deproteinized Calf Blood Extract Injection (96 h, n = 4).**

| Group | 1 | |  | 2 | |  | 3 | |  | 4 | |
| --- | --- | --- | --- | --- | --- | --- | --- | --- | --- | --- | --- |
|  | Left Side | Right Side |  | Left Side | Right Side |  | Left Side | Right Side |  | Left Side | Right Side |
| Single IM Injection | 0 | 0 |  | 0 | 0 |  | 0 | 0 |  | 0 | 0 |
| Repeated IM Injections (×6) | 0 | 0 |  | 0 | 0 |  | 0 | 0 |  | 0 | 0 |

Note: The Left Side served as the experimental site and received 0.5 mL of undiluted Deproteinized Calf Blood Extract Injection, while the Right Side served as the control site and received 0.5 mL of 0.9% Sodium Chloride Injection.

**Supplementary Table S5. Results of Intravenous Administration of Deproteinized Calf Blood Extract Injection in Mice.**

| **Dose of Deproteinized Calf Blood Extract Injection (mg·kg-1)** | **Number of Animals** | **Number of Deaths** |
| --- | --- | --- |
| 6150 | 10 | 10 |
| 5169 | 10 | 8 |
| 4346 | 10 | 6 |
| 3650 | 10 | 1 |
| 3075 | 10 | 0 |

**Supplementary Table S6.** Allergic Reaction Assessment Table.

| **Reaction Symptom** | **Reaction Grade** |
| --- | --- |
| No obvious reaction | 0 |
| Mild nose-scratching, shivering, or piloerection | 1 |
| Several coughs, nose-scratching, shivering, or piloerection | 2 |
| Repeated or continuous coughing with dyspnea, spasm, or convulsion | 3 |
| Spasm, convulsion, urinary/fecal incontinence, or shock/death | 4 |

**Supplementary Table S7.** Sample Addition Scheme for In Vitro Hemolysis Test of Deproteinized Calf Blood Extract Injection.

| Tube No. | **1** | **2** | **3** | **4** | **5** | **6** | **7** |
| --- | --- | --- | --- | --- | --- | --- | --- |
| 2% Erythrocyte Suspension (mL) | 2.5 | 2.5 | 2.5 | 2.5 | 2.5 | 2.5 | 2.5 |
| 0.9% Sodium Chloride Solution (mL) | 2.0 | 2.1 | 2.2 | 2.3 | 2.4 | 2.5 | - |
| Distilled Water (mL) | - | - | - | - | - | - | 2.5 |
| DCBEI (mL) | 0.5 | 0.4 | 0.3 | 0.2 | 0.1 | - | - |

**Supplementary Table S8.** Scoring Criteria for Skin Irritation Reactions.

| **Erythema Reactions** | **Score** | **Edema Reactions** | **Score** |
| --- | --- | --- | --- |
| No erythema | 0 | No edema | 0 |
| Barely perceptible | 1 | Barely perceptible | 1 |
| Clearly visible | 2 | Visible (edges elevated above surrounding skin) | 2 |
| Severe erythema | 3 | Skin elevated ≈1 cm with well-defined contour | 3 |
| Dark red erythema with scab formation | 4 | Edema elevated >1 cm with extended area | 4 |

**Supplementary Table S9.** Criteria for Evaluation of Skin Irritation Intensity.

| Mean Score | Evaluation |
| --- | --- |
| 0~0.49 | No irritation |
| 0.5~2.99 | Mild irritation |
| 3.0~5.99 | Moderate irritation |
| 6.0~8.0 | Severe irritation |

**Supplementary Table S10.** Criteria for Evaluation of Muscular Irritation Reactions.

| **Irritation Reaction** | **Reaction Grade** |
| --- | --- |
| No obvious reaction at injection site | 0 |
| Mild congestion at injection site, diameter < 0.5 cm | 1 |
| Moderate congestion at injection site, diameter < 1.0 cm | 2 |
| Severe congestion with erythema and muscular degeneration | 3 |
| Brownish muscular degeneration with necrosis, diameter < 0.5 cm | 4 |
| Severe muscular degeneration with extensive necrosis | 5 |

**Supplementary Table S11.** Body weight changes (x ± s) in mice after intravenous administration of Deproteinized Calf Blood Extract Injection.

| **Group** | **Body Weight (g)** | | | |
| --- | --- | --- | --- | --- |
|  | **Before Administration** | **Day 5** | **Day 10** | **Day 15** |
| 6150 mg·kg^-1^ | 20.0±1.49 | 23.0±2.83 | 27.0±4.24 | 31.0±4.24 |
| 5169 mg·kg^-1^ | 20.2±0.92 | 24.0±1.41 | 27.8±0.96 | 32.8±1.50 |
| 4346 mg·kg^-1^ | 20.2±1.32 | 24.1±1.05 | 28.3±1.12 | 33.0±1.41 |
| 3650 mg·kg^-1^ | 20.4±1.07 | 24.4±1.35 | 28.6±1.35 | 33.4±1.26 |
