# Supplementary figures and images for: Local and systemic safety of deproteinized calf blood extract injection: hypersensitivity, hemolysis, local tolerance, and acute intravenous toxicity in rodents and rabbits
Source: Front Pharmacol. 2026 Jan 5;16:1709084. doi: 10.3389/fphar.2025.1709084 (PMC12813156; doi:10.3389/fphar.2025.1709084)

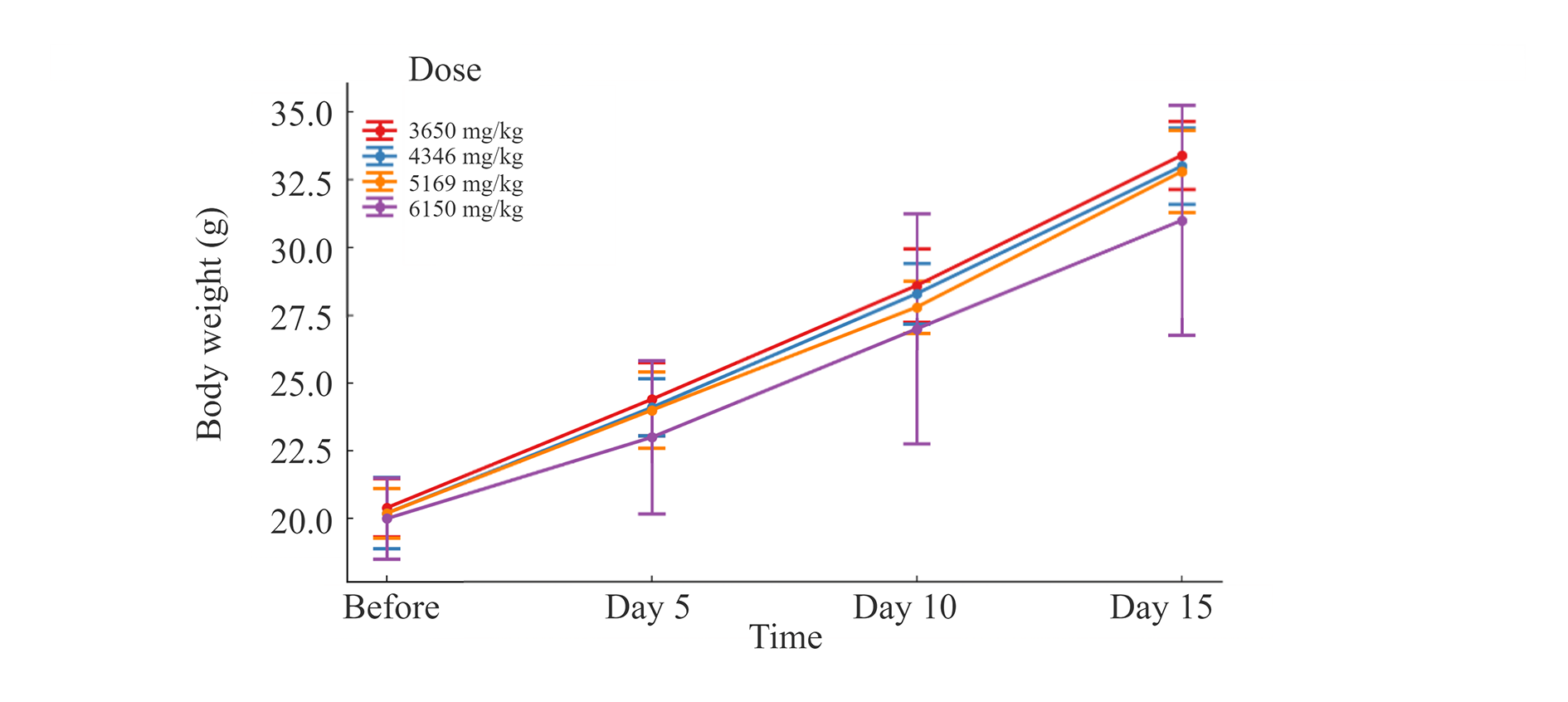

Supplement: Supplementary file 2 [file Image1.tif]
